# Supplementary material for: Chromothripsis during telomere crisis is independent of NHEJ, and consistent with a replicative origin
Source: Genome Res. 2019 May;29(5):737–49. doi: 10.1101/gr.240705.118 (PMC6499312; doi:10.1101/gr.240705.118)
Supplement: Supplemental Material [file supp_gr.240705.118_Supplemental_file_1.zip › contigs/annotated_contigs/DB114/contig.3.DB114_length_405_mean_cov_11.1209876543.docx]

**DB114_length_405_mean_cov_11.1209876543**

```
TAACCAAATTATTGTGAATTAAAATTCACAACTTATGGCATTAAATCAAAGATTACCAGGCATGCACAGAAATAGAAA|AAC|TTGTGA
 >chr20:62016290-62016371 + E=3e-37                                            >chr20:6
CCTCAAGTGATCCACCCACCTTGGCCTCCCAAAGTGCTGGGATTACAGGGATGAGCCACCACACCCAACCTCTGATAACATTTGAAATA
2016336-62016537 - E=2e-109
TCCTTGTCTATTACCTCTACATCTACATCAGTTTGGGGTCAGTTTCAATCAATTGATTTGGGGGCCACGTTTTTCTATTTCTGTGCATG

CCTGGTAATCTTTG|GCC|GCTGGATGCTGAATAAGCCAGGAAGAAGATCCGGCTAAATGTTGGCACATTCTAAAGTCTACGTGAGGCC
                  >chr20:62017643-62017766 + E=2e-62
AGTCTGACCCTGGGAACCTCCATGAAGACATGGGCGTGGAGGGTCTGCCTTTT
```
